# The severity of Puumala hantavirus induced nephropathia epidemica can be better evaluated using plasma interleukin-6 than C-reactive protein determinations

**DOI:** 10.1186/1471-2334-10-132

**Published:** 2010-05-25

**Authors:** Tuula K Outinen, Satu M Mäkelä, Ilpo O Ala-Houhala, Heini SA Huhtala, Mikko Hurme, Antti S Paakkala, Ilkka H Pörsti, Jaana T Syrjänen, Jukka T Mustonen

**Affiliations:** 1 Department of Internal Medicine, Tampere University Hospital, P.O.Box 2000, Tampere, FI-33521, Finland; 2 Medical School, University of Tampere, Tampere, FI-33014, Finland; 3 Tampere School of Public Health, University of Tampere, Tampere, FI-33014, Finland; 4 Department of Radiology, Tampere University Hospital, P.O. Box 2000, Tampere, FI-33521, Finland

## Abstract

**Background:**

Nephropathia epidemica (NE) is a Scandinavian type of hemorrhagic fever with renal syndrome caused by Puumala hantavirus. The clinical course of the disease varies greatly in severity. The aim of the present study was to evaluate whether plasma C-reactive protein (CRP) and interleukin (IL)-6 levels associate with the severity of NE.

**Methods:**

A prospectively collected cohort of 118 consecutive hospital-treated patients with acute serologically confirmed NE was examined. Plasma IL-6, CRP, and creatinine, as well as blood cell count and daily urinary protein excretion were measured on three consecutive days after admission. Plasma IL-6 and CRP levels higher than the median were considered high.

**Results:**

We found that high IL-6 associated with most variables reflecting the severity of the disease. When compared to patients with low IL-6, patients with high IL-6 had higher maximum blood leukocyte count (11.9 *vs *9.0 × 10^9^/l, *P *= 0.001) and urinary protein excretion (2.51 *vs *1.68 g/day, *P *= 0.017), as well as a lower minimum blood platelet count (55 *vs *80 × 10^9^/l, *P *< 0.001), hematocrit (0.34 *vs *0.38, *P *= 0.001), and urinary output (1040 *vs *2180 ml/day, *P *< 0.001). They also stayed longer in hospital than patients with low IL-6 (8 *vs *6 days, *P *< 0.001). In contrast, high CRP did not associate with severe disease.

**Conclusions:**

High plasma IL-6 concentrations associate with a clinically severe acute Puumala hantavirus infection, whereas high plasma CRP as such does not reflect the severity of the disease.

## Background

Nephropathia epidemica (NE) is a Scandinavian type of hemorrhagic fever with renal syndrome. The causative agent, Puumala virus (PUUV), is a member of the genus *Hantavirus *in the family *Bunyaviridae *[1]. Other hantaviruses causing more severe forms of HFRS include Hantaan, Seoul, and Dobrava viruses [2]. Many hantaviruses in North and South America, e.g. Sin Nombre, Andes, and Black Creek Canal viruses, cause hantavirus pulmonary syndrome (HPS) [2]. The natural host of PUUV is the bank vole (*Myodes glareolus*) [3].

Nephropathia epidemica is prevalent in Finland, elsewhere in Scandinavia, in Western Russia, in the Balkan region and also in many parts of Western Europe [2,4]. Approximately 1000 serological diagnoses of PUUV infection are made in Finland annually [5]. However, the seroprevalence in the Finnish population is 5%, implying that most infections are subclinical or undiagnosed [5].

The clinical severity of NE varies greatly. Host genetics have been shown to influence the clinical picture [6,7]. It is clinically characterized by acute fever, headache, back and abdominal pains, myalgia, nausea, vomiting, and transient myopia, while hemorrhages are uncommon [8,9]. Renal involvement causes proteinuria, hematuria and oliguria, followed by polyuria [8,9]. A minority of patients needs transient dialysis treatment [8], but complete recovery is the usual outcome [8,9]. During the acute phase, an increase in the serum creatinine concentration, thrombocytopenia, anemia, leukocytosis, as well as moderately elevated erythrocyte sedimentation rate and C-reactive protein (CRP) values are typical laboratory findings [8,9]. In addition, radiological pulmonary manifestations have been detected in 16-53% of the patients, and they have been associated with the degree of acute renal insufficiency [8-12].

The pathogenesis of NE is not completely understood. An important feature in hantavirus infections is universally increased capillary permeability [13], but the mechanisms of vascular leakage are unclear. PUUV causes no cytopathic effects in cultured cells but has a wide cell susceptibility *in vitro *[14]. It has been suggested that immunological factors including cytokines are involved in the pathogenesis of NE [2]. Increased cytokine levels have been found in the plasma, urine, and tissues of hantavirus infection patients [15-18]. Infection of cynomolgus macaques by PUUV also results in increased serum levels of several cytokines [19]. We have previously found plasma and urinary levels of interleukin (IL)-6, tumor necrosis factor-α, IL-1, and IL-1-receptor antagonist to be increased during the acute phase of NE, so that the observed levels of IL-6 were exceptionally high [15].

The IL-6 molecule is a multifunctional cytokine involved in immune responses and inflammation. IL-6 is the main inducer of CRP production *in vitro *in cultured human hepatoma cells [20], but data about the associations of IL-6 and CRP *in vivo *is scarce. The known main functions of CRP are complement activation, enhancement of phagocytosis, and induction of cytokine synthesis. Although plasma CRP level is widely used as an indicator of the severity of the disease in various infections, there are no reports associating high CRP levels to a severe disease in NE or other viral infections. On the other hand, IL-6 level has been found to be associated to the severity of the disease in some viral infections, e.g. influenza [21,22]. Therefore, we studied whether plasma IL-6 or CRP levels are associated with the severity of NE, in order to evaluate if IL-6 or CRP are good markers for disease severity in NE.

## Methods

### Patients

The study cohort originally consisted of 131 prospectively collected consecutive patients with acute NE treated at Tampere University Hospital, Finland, between September 1997 and December 2004. We have previously studied urinary IL-6 excretion in 70 NE patients treated during the years 1997-1999 [15], these patients were also included in the present material. Now plasma IL-6 levels were measured altogether from 118 patients, who comprised the final cohort of the present study, as we did not have plasma samples for IL-6 determinations from 13 subjects. The median patient age was 40 (ranging 15-71) years, and 86 (73%) were males.

Thirty-seven (31%) patients had one or more of the following diseases before NE: essential hypertension in 10, dyslipidemia in six, hypothyreosis in five, coronary artery disease in four, and bronchial asthma in three; atrial fibrillation, celiac disease, chronic inflammatory bowel disease, and hyperplasia of the prostate in two; sick sinus syndrome treated with pacemaker, diabetes mellitus, osteoporosis, ankylosing spondylarthritis, aortic valve disease, mitral valve disease, epilepsy, fibromyalgia, sarcoidosis, multiple sclerosis, operated melanoma, operated cancer of vocal cords, operated meningeoma, and sequelae of renal tuberculosis in one patient each.

The diagnosis of acute PUUV infection was serologically confirmed in all cases [23]. All subjects gave informed consent before participation and the study was approved by the Ethics Committee of Tampere University Hospital.

### Study protocol

All 118 patients were studied during the acute phase of NE. A detailed past and current medical history was obtained, and a careful physical examination was performed. Blood samples to analyze plasma IL-6 and CRP, serum creatinine, and blood cell count, as well as daily urinary protein excretion were collected on three consecutive mornings after hospital admission. Other blood samples were taken according to the clinical needs of the patient. The highest and the lowest value of each patient of the various variables measured during hospitalization were designated as the maximum and minimum values. In this study, we have defined high serum creatinine as a value exceeding the median maximum creatinine among the study population (193 μmol/l) and thrombocytopenia as the minimum platelet count equal to or lower than the median among the study population (66 × 10^9^/l). High plasma CRP was defined as the maximum CRP value above the median in the study population (69 mg/l) and high plasma IL-6 as the maximum IL-6 value higher than 14.05 pg/ml (the median in the study population).

### Methods

All blood specimens were obtained between 7:30-9:30 in the morning. Plasma CRP was analyzed by Hitachi 705 E Analyzer from 1997 to 1998 and after that by the Roche Diagnostics CRP method using Cobas Integra analyzer (F. Hoffman-La Roche Ltd, Basel, Switzerland). Blood cell count was completed by hematological cell counters by Bayer. From 1997 to June 1999, serum creatinine was determined by Vitros (Johnson & Johnson, Rochester, NY, USA) and after that by Cobas Integra analyzer. Serum creatinine concentrations showed 10% lower values after June 1999 than during the earlier years due to the change in the determination method. Therefore, in this study the results of serum creatinine concentrations from September 1997 to June 1999 were multiplied by the coefficient 0.9. Plasma IL-6 concentrations were determined afterwards from frozen samples by using commercially available enzyme-linked immunosorbent assays (PeliKine Compact human IL-6 kits; Central Laboratory of the Netherlands, Red Cross Blood Transfusion Service, Amsterdam, The Netherlands), following the manufacturer's instructions. Detection limit for the assay was 0.4 pg/ml for IL-6. The patients in this study did not have values below the detection limit. The 24-hour urinary protein excretion was measured by the pyrogallolal red molybdate method (Olli C.; Kone Instruments, Helsinki, Finland) from 1997 to April 1998 and after that by Cobas Integra analyzer, from a total of 72 patients. One chest radiograph was obtained during hospitalization from 77 patients, two from 16 and three from two patients.

### Statistical Analysis

In order to describe the data, medians (ranging) were given for continuous variables and numbers and percentages for categorical variables.

To evaluate the associations of plasma IL-6 and CRP values with the severity of NE, we divided the patients into two groups, first according to the maximum IL-6 value and then according to the maximum CRP value. For the purposes of further evaluating the effect of plasma IL-6 and plasma CRP on the severity of the disease, we divided the patients into four groups: group 1 with low IL-6 and low CRP (equivalent to or lower than the median), group 2 with low IL-6 and high CRP (higher than the median), group 3 with high IL-6 and low CRP, and group 4 with both high IL-6 and high CRP.

Groups were compared using the Mann-Whitney *U- *test or Kruskal-Wallis test, as appropriate. Categorical data were analyzed by the *x*^2 ^test or the Fisher's exact test. Correlations were calculated by means of Spearman's rank correlation coefficient. We also performed logistic regression analyses with high serum creatinine, thrombocytopenia, or hospitalization exceeding seven days as dependent factors and high plasma IL-6 and high plasma CRP as independent factors in order to further examine the association of these factors with the severity of the disease. Age was also included in these models as a continuous variable. Adjusted odds ratios (OR) and their 95% confidence intervals (95% CI) were given. All tests were two-sided, and statistically significant *P*-values are given. All analyses were made with the SPSS (version 7.5) statistical software package.

## Results

The clinical and laboratory characteristics of the patients are shown in Table 1. Three (3%) of the total 118 patients were in clinical shock at admission, and six (5%) required dialysis treatment during hospital care. Thirty-four of the patients (29%) had a plasma CRP value higher than 100 mg/l, 59 (50%) had a leukocyte count higher than 10.0 × 10^9^/l, 34 (29%) had a platelet count lower than 50 × 10^9^/l, and 88 (75%) had a serum creatinine value higher than 100 μmol/l during the hospital stay. Thirty patients (32%) presented with pathologic findings in a chest radiograph. Everyone recovered completely. The median duration of fever before admission to the hospital was four (ranging 1-14) days.

**Table 1:** The clinical and laboratory findings in 118 patients with acute Puumala hantavirus infection.

Symptoms and findings	Median	Ranging
Duration of fever (days)	5	2-15
Duration of hospital stay (days)	7	2-15
SBP min (mmHg)	112	82-162
Change in weight during hospital stay (kg)	2.6	0-12.0
Urinary output min (ml/day)	1520	50-7000
Urinary protein max (g/day)	1.80	0.14-17.78
Creatinine max (μmol/l)	193	65-1285
Platelets min (10^9^/l)	66	3-238
Hematocrit min	0.36	0.25-0.46
Leukocytes max (10^9^/l)	10.0	3.9-31.2
CRP max (mg/l)	69	11-269
IL-6 max (pg/ml)	14.05	1.31-107.00

SBP = systolic blood pressure, min = minimum, max = maximum, CRP = C-reactive protein, IL-6 = interleukin-6.

The median age did not differ between patients with high plasma IL-6 and patients with low IL-6 (41 years, ranging 15-65 *vs *39 years, ranging 17-71, *P *= 0.741). Forty-four (75%) of the patients with high IL-6 were males compared with 42 (71%) of the patients with low IL-6 (*P *= 0.679). In patients with high plasma CRP, the median age was higher than in patients with low CRP (46 years, ranging 25-71 *vs *38 years, ranging 15-64, *P *< 0.001). Forty-two (72%) of the patients with high CRP were males compared with 44 (73%) of the patients with low CRP (*P *= 0.911).

**Figure 1:**
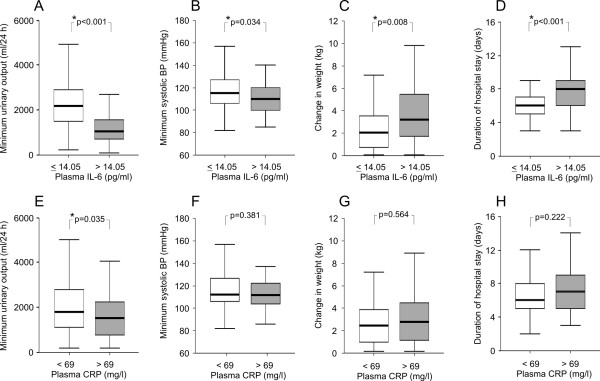
**The associations of various clinical variables with maximum plasma interleukin-6 and C-reactive protein concentrations in patients with Puumala hantavirus infection**. Data are given as median (thick line), 25^th^-75^th ^percentile (box), and range (whiskers); outliers have been omitted from the figure.

**Figure 2:**
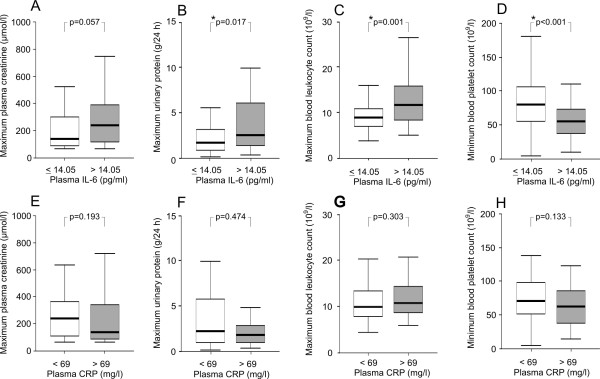
**The associations of laboratory variables with maximum plasma interleukin-6 and C-reactive protein concentrations in patients with Puumala hantavirus infection**. Data are given as median (thick line), 25^th^-75^th ^percentile (box), and range (whiskers); outliers have been omitted from the figure.

The maximum level of plasma IL-6 associated strongly with several variables reflecting the severity of the disease (Figures 1 and 2). Patients who had high IL-6 had lower minimum urinary output (1040 *vs *2180 ml/day, Figure 1A), lower minimum systolic blood pressure (110 *vs *115 mmHg, Figure 1B), and a greater change in weight during hospital care (3.2 *vs *2.0 kg, Figure 1C), and they also stayed longer in hospital than patients with low IL-6 (8 *vs *6 days, Figure 1D). Furthermore, patients with high IL-6 had numerically (but not statistically significantly) higher maximum serum creatinine levels (242 *vs *140 μmol/l, Figure 2A), higher maximum urinary protein excretion (2.51 *vs *1.68 g/day, Figure 2B), higher maximum leukocyte count (11.9 *vs *9.0 × 10^9^/l, Figure 2C), lower minimum platelet count (55 *vs *80 × 10^9^/l, Figure 2D), and lower minimum hematocrit (0.34 *vs *0.38, *P *= 0.001) than patients with low IL-6. The clinical or laboratory variables between the patients with high and low CRP did not differ (Figures 1F-H, 2E-H) with the exception of minimum urinary output. Patients with high CRP had slightly lower minimum urinary output when compared to patients with low CRP (1400 *vs *1700 ml/day, Figure 1E).

The occurrence of a pathologic chest radiograph had no significant associations with high IL-6 or high CRP. Twenty patients (38%) with high IL-6 had a pathologic chest x-ray compared with 10 patients (24%) with low IL-6 (*P *= 0.147). Eighteen patients (37%) with high CRP had a pathologic chest x-ray compared with 12 patients (26%) with low CRP (*P *= 0.265).

There was a positive correlation between plasma CRP and plasma IL-6 levels (r = 0.323, *P *< 0.001). We also found a slight positive correlation between maximum plasma IL-6 and serum creatinine concentrations (r = 0.238, *P *= 0.010), whereas no correlation was found between maximum plasma CRP and serum creatinine levels.

Tables 2 and 3 show the associations of various variables reflecting the severity of the disease with plasma IL-6 and CRP levels in patients divided into four groups according to maximum IL-6 and CRP values. Table 2 shows that especially patients with an IL-6 value higher than the median, with or without high CRP (groups 3 and 4), had more severe disease as measured with several clinical parameters. Table 3 shows the occurrence of shock, dialysis treatment, pathologic findings in a chest x-ray, high serum creatinine, thrombocytopenia, and hospitalization longer than seven days (median) in these four groups. Significantly fewer patients in group 2 (low IL-6 and high CRP) had high serum creatinine levels compared to the other three groups (group 2 *vs *group 1, *P *= 0.048; group 2 *vs *group 3, *P *= 0.002; group 2 *vs *group 4, *P *= 0.024). Furthermore, none of the patients in group 2 required dialysis treatment.

**Table 2:** The clinical and laboratory variables in patients with Puumala hantavirus infection divided into four groups according to maximum plasma interleukin-6 and C-reactive protein levels.

	Group 1 (N = 37) CRP ≤ 69 mg/l IL-6 ≤ 14.05 pg/ml	Group 2 (N = 22) CRP > 69 mg/l IL-6 ≤ 14.05 pg/ml	Group 3 (N = 23) CRP ≤ 69 mg/l IL-6 > 14.05 pg/ml	Group 4 (N = 36) CRP > 69 mg/l IL-6 > 14.05 pg/ml	P-value
Age (years)	38 (17-63)	51 (26-71)	36 (15-64)	45 (25-65)	0.013
Hospital stay (days)	6 (2-15)	6 (3-10)	7 (4-13)	8 (3-14)	0.003
SBP min (mmHg)	117 (82-162)	112 (93-149)	110 (85-140)	112 (86-158)	0.092
Change in weight (kg)	2.1 (0-9.9)	1.3 (0-4.7)	2.8 (0-12.0)	3.3 (0-9.9)	0.023
Urinary output min (ml/day)	2230 (350-7000)	2050 (200-4940)	1040 (50-4900)	1045 (50-3325)	< 0.001
Urinary protein max (g/day)	1.43 (0.14-5.59)	2.03 (0.57-4.31)	6.05 (0.30-10.00)	1.72 (0.30-17.78)	0.003
Creatinine max (μmol/l)	193 (65-917)	114 (68-878)	256 (88-1285)	230 (70-1156)	0.073
Platelets min (10^9^/l)	80 (3-238)	84 (19-159)	59 (9-139)	54 (13-187)	0.001
Hematocrit min	0.38 (0.29-0.43)	0.38 (0.29-0.44)	0.34 (0.25-0.43)	0.35 (0.25-0.46)	0.007
Leukocytes max (10^9^/l)	9.0 (3.9-31.2)	9.2 (5.4-18.6)	11.0 (5.1-23.2)	12.2 (7.1-26.8)	0.011

Values are expressed as medians (ranging). SBP = systolic blood pressure, min = minimum, max = maximum, CRP = plasma C-reactive protein, IL-6 = plasma interleukin-6.

**Table 3:** Categorical variables associated with the severity of the disease in nephropathia epidemica patients, divided into four groups according to maximum plasma interleukin-6 and C-reactive protein levels.

	Group 1 (N = 37) CRP ≤ 69 mg/l IL-6 ≤ 14.05 pg/ml	Group 2 (N = 22) CRP > 69 mg/l IL-6 ≤ 14.05 pg/ml	Group 3 (N = 23) CRP ≤ 69 mg/l IL-6 > 14.05 pg/ml	Group 4 (N = 36) CRP > 69 mg/l IL-6 > 14.05 pg/ml	P-value
Gender (males)	28 (76%)	14 (64%)	16 (70%)	28 (78%)	0.645
Shock	0 (0%)	0 (0%)	1 (4%)	2 (6%)	
Dialysis treatment	1 (3%)	0 (0%)	2 (9%)	3 (8%)	
Pathologic chest x-ray	6 (23%)	4 (25%)	6 (30%)	14 (42%)	0.390
Hospital stay > 7 days	7 (19%)	5 (23%)	11 (48%)	19 (53%)	0.007
Creatinine max > 193 μmol/l	18 (49%)	5 (23%)	16 (70%)	19 (53%)	0.017
Platelets min ≤ 66 × 10^9^/l	14 (38%)	7 (32%)	13 (57%)	27 (75%)	0.002

Values are expressed as numbers and percentages. CRP = plasma C-reactive protein, IL-6 = plasma interleukin-6, max = maximum, min = minimum.

Logistic regression analyses were then carried out to evaluate the associations of high CRP and high IL-6 with high serum creatinine, thrombocytopenia, or hospitalization exceeding seven days. High plasma IL-6 was revealed as an independent risk factor for high serum creatinine (OR 3.2, 95% CI 1.4-7.3, *P *= 0.005), whereas high plasma CRP was found to be a protective factor (OR 0.3, 95% CI 0.1-0.7, *P *= 0.009) in this model. High plasma IL-6 was also found to be an independent risk factor for thrombocytopenia and hospitalization exceeding seven days (OR 3.6, 95% CI 1.6-8.0, *P *= 0.002, and OR 4.5, 95% CI 1.9-10.8, *P *< 0.001, respectively) in models including high plasma IL-6, high plasma CRP, and age. High plasma CRP did not have a significant association with these factors (data not shown).

As the patients with NE sought medical assistance at different time intervals from the onset of fever, the plasma CRP and IL-6 samples were also taken at different periods from the onset. From the majority of patients (66%) we had both CRP and IL-6 samples taken 5 days from the onset of fever. In this subgroup of 78 patients the main results remained the same: when compared to patients with low IL-6, patients with high IL-6 had higher creatinine levels, higher leukocyte count, greater change in weight, as well as a lower platelet count, hematocrit, and urinary output. They also stayed longer in hospital than patients with low IL-6. High CRP five days after the onset of fever had no associations with the variables reflecting the severity of NE (data not shown).

## Discussion

The present study with 118 consecutive prospectively collected hospitalized patients is by far the largest study concerning inflammatory parameters, i.e. IL-6 and CRP, in acute Puumala hantavirus infection. The present data showed that high plasma IL-6 is associated with clinically severe NE. High IL-6 was found to be an independent risk factor for impaired renal function, thrombocytopenia, and longer hospitalization, when examined together with high CRP and age. Surprisingly, the results also suggested that high plasma CRP might have a protective effect on renal function, but the data must be interpreted with caution.

We found that maximum plasma IL-6 was associated with the severity of renal insufficiency, blood leukocytosis and thrombocytopenia. It also associated strongly with the duration of hospitalization. We have previously studied plasma IL-6 concentrations in a cohort of 70 NE patients and found IL-6 levels to be increased [15]. In that earlier study, there was no correlation between plasma IL-6 levels and serum creatinine, but in the present larger study we did find a correlation between the levels of these two variables.

Previously, Linderholm and co-workers have studied 15 NE patients and detected elevated IL-6 plasma levels in all cases [17]. They also found a significant correlation between maximum levels of IL-6 and serum creatinine in concordance with our results. Takala and co-workers have studied 19 NE patients and 13 patients with other viral infections and detected an inverse correlation of serum IL-6 concentrations in NE patients with mean arterial pressure and minimum platelet count [24]. Plasma levels of IL-6 have been reported to associate with the severity of the disease also in influenza and Japanese encephalitis [21,22,25]. Studies of IL-6 in respiratory syncytial virus infection and Dengue virus infection have produced controversial results [26-29]. In acute renal failure, it has been demonstrated that plasma IL-6 levels are elevated and that high levels predict mortality [30,31].

As far as we know, this is the first report suggesting that CRP might act as a protective factor for renal function in infectious diseases. Previously, it has been shown in mice that CRP prevents and reverses proteinuria in accelerated nephrotoxic nephritis [32-34]. It has also been reported that genetics associated with reduced CRP production predispose to the development of systemic lupus erytematosus [35]. This has been attributed to the ability of CRP to prevent the deposition of immune complexes and enhance their phagocytosis. It should be noted that immune complexes have also been found in NE patients [36]. Reduced deposition and enhanced phagocytosis of immune complexes could be the mechanism by which high CRP protects renal function in NE. In viral infections, there are no reports of CRP concentrations affecting the severity of the disease. In bacteremia and sepsis, the results concerning CRP as a predictor of clinical outcome are controversial. A year 2005 review concludes that the ability of CRP level to reflect the severity of sepsis may be limited [37].

Finally, there was a positive correlation between maximum IL-6 and CRP levels in the present study, which can be explained by the fact that IL-6 induces the production of CRP. However, high IL-6 concentration was associated with more severe disease, whereas high CRP level, in contrast, was associated with less severe renal impairment. This finding can probably be explained by the diverse biological influences of IL-6 and CRP.

## Conclusions

High plasma IL-6 concentration is associated with clinically severe acute NE and could be used as a marker of the severity of the disease. On the other hand, high CRP as such does not indicate a severe form of NE.

## Competing interests

The authors declare that they have no competing interests.

## Authors' contributions

TO has written the manuscript and analysed the data. SM has recruited and examined the patients as well as designed and organized the study. IA-H has recruited and examined the patients. HH has checked the statistics to be correct. MH has determined the IL-6 levels. AP has interpreted the x-rays. IP has designed and organized the study and helped with the figures. JS has designed and organized the study. JM has designed and organized the study. All authors have been involved in revising the manuscript critically and have given final approval of the version to be published.

## Pre-publication history

The pre-publication history for this paper can be accessed here:

http://www.biomedcentral.com/1471-2334/10/132/prepub
